# A Comprehensive Self-Management Intervention for Inflammatory Bowel Disease (CSM-IBD): Protocol for a Pilot Randomized Controlled Trial

**DOI:** 10.2196/46307

**Published:** 2023-06-07

**Authors:** Kendra Kamp, Kindra Clark-Snustad, Linda Yoo, Samantha Winders, Kevin Cain, Rona L Levy, Neelendu Dey, Scott Lee, Laurie Keefer, Margaret Heitkemper

**Affiliations:** 1 Department of Biobehavioral Nursing and Health Informatics School of Nursing University of Washington Seattle, WA United States; 2 Division of Gastroenterology School of Medicine University of Washington Seattle, WA United States; 3 School of Social Work University of Washington Seattle, WA United States; 4 Fred Hutchinson Cancer Center Seattle, WA United States; 5 Mount Sinai New York, NY United States

**Keywords:** inflammatory bowel disease, self-management, symptom management, pilot, intervention, randomized controlled trial

## Abstract

**Background:**

Despite pharmacological treatment, individuals with inflammatory bowel disease (IBD) experience a variety of symptoms, including abdominal pain, fatigue, anxiety, and depression. Few nonmedical self-management interventions are available for people with IBD. A validated comprehensive self-management (CSM) intervention is effective for patients with irritable bowel syndrome who can have symptoms similar to those of individuals with IBD. We created a modified CSM intervention tailored to individuals with IBD (CSM-IBD). The CSM-IBD is an 8-session program delivered over 8-12 weeks with check-ins with a registered nurse.

**Objective:**

The primary objective of this pilot study is to determine the feasibility and acceptability of study procedures and the CSM-IBD intervention and to evaluate preliminary efficacy on quality of life and daily symptoms for a future randomized controlled trial. Additionally, we will examine the association of socioecological, clinical, and biological factors with symptoms at baseline and response to intervention.

**Methods:**

We are conducting a pilot randomized controlled trial of the CSM-IBD intervention. Participants aged 18-75 years who are experiencing at least 2 symptoms are eligible for inclusion. We plan to enroll 54 participants who will be randomized (2:1) into the CSM-IBD program or usual care. Patients in the CSM-IBD program will have 8 intervention sessions. Primary study outcomes include the feasibility of recruitment, randomization, and data or sample collection, as well as the acceptability of study procedures and interventions. Preliminary efficacy outcome variables include quality of life and symptoms. Outcomes data will be assessed at baseline, immediately post intervention, and 3 months post intervention. Participants in the usual care group will have access to the intervention after study participation.

**Results:**

This project is funded by the National Institutes of Nursing Research and reviewed by the University of Washington’s institutional review board. Recruitment began in February 2023. As of April 2023, we have enrolled 4 participants. We expect the study to be completed by March 2025.

**Conclusions:**

This pilot study will evaluate the feasibility and efficacy of a self-management intervention (a web-based program with weekly check-ins with a registered nurse) that aims to improve symptom management in individuals with IBD. In the long term, we aim to validate a self-management intervention to improve patient quality of life, reduce direct and indirect costs related to IBD, and be culturally appropriate and accessible, particularly in rural and underserved communities.

**Trial Registration:**

ClinicalTrials.gov NCT05651542; https://clinicaltrials.gov/ct2/show/NCT05651542

**International Registered Report Identifier (IRRID):**

PRR1-10.2196/46307

## Introduction

### Background and Rationale

Inflammatory bowel disease (IBD; including ulcerative colitis and Crohn disease) is a chronic immune-mediated disease of the gastrointestinal system that impacts individuals worldwide. Patients with IBD are often diagnosed at a young age and experience a lifetime of disease management, with symptoms including diarrhea, pain, fatigue, urgency, anxiety, and depression [1,2]. These symptoms result in suffering, substantial health care costs, and indirect costs related to work and school impairment, with fatigue being the most common reason for work absenteeism [3]. Health care providers are often not trained to manage noninflammatory symptoms, and with improved medications such as biologics and small molecule therapy, the disconnect between patient symptoms and management is widening [4,5]. Our team has a comprehensive solution to help patients with irritable bowel syndrome manage symptoms and improve self-management skills that can be adapted for an IBD population to address the urgent need for symptom management.

Within the United States, approximately 3.1 million individuals have a diagnosis of IBD [6]. As societies become more westernized, IBD incidence and prevalence have increased worldwide [7]. IBD affects men and women equally, although ulcerative colitis is slightly more common in males and Crohn disease is slightly more frequent in females [8]. In the past, it was thought that IBD occurred less frequently in racial and ethnic minorities compared with whites; however, this gap is narrowing with an increased incidence of IBD among African American and Asian individuals [9]. Therefore, with growing incidence and prevalence worldwide and among racial and ethnic minority populations, IBD represents a growing health problem demanding tailored, culturally appropriate, and effective interventions.

Immunosuppressive therapy can be effective in achieving control of endoscopic inflammation from IBD; yet, patients can experience symptoms while they await response to therapy or in the setting of refractory disease [10,11]. Patients can also have chronic symptoms, even after achieving remission, due to damage from chronic disease, altered surgical anatomy, or co-occurring functional symptoms [12]. While immunosuppression is essential in achieving and maintaining endoscopic remission for patients with moderate-severe IBD, other interventions are needed to improve symptom management and quality of life.

Symptom-directed medical therapies (eg, antidiarrheals, antiemetics, antispasmodics, and neuromodulators) offer symptom relief for some but are not effective for everyone and offer limited benefit for symptoms such as pain and fatigue. Nonpharmacological interventions such as dietary modification, relaxation techniques, and cognitive behavioral therapy (CBT) have been shown to improve symptoms [13,14], but teaching patients how to implement these interventions is time-consuming and impractical to achieve in a routine clinic visit. Some clinics have used an IBD specialty medical home model, which can reduce hospitalizations and emergency department visits as well as increase the quality of life [15]. Yet, patient access to providers who are experienced in these interventions and care delivery systems can be limited, especially in rural and underserved communities [16,17].

As the majority of a patient’s time is spent outside of the clinic, there is a need to empower patients with the skills to manage their disease. Self-management refers to the day-to-day management that individuals must engage in while living with a chronic condition [18,19]. Among individuals with other chronic conditions, self-management programs are effective in reducing hospitalizations, days in the hospital, and fatigue while increasing self-reported health [20,21]. Despite the effectiveness of self-management programs among other chronic condition populations, few effective self-management interventions exist for individuals with IBD [22]. A systematic review identified 6 self-management randomized controlled trials conducted among individuals with IBD (representing 1715 patients) [22]. However, only half of the interventions improved health-related quality of life or reduced disease activity at 12 months. Psychological outcomes were reported by 2 studies, and 1, which used a written self-management plan developed with a clinician, failed to demonstrate an improvement in depression and anxiety [23,24].

An existing comprehensive self-management (CSM) intervention that provides patient education on nonmedical interventions for symptom management (eg, dietary modification, stress reduction, and CBT) has been shown to improve quality of life and decrease abdominal pain in patients with irritable bowel syndrome [25-27]. Furthermore, the intervention reduced anxiety, depression, fatigue, and extraintestinal pain and improved self-reported sleep compared to usual care [28,29]. To date, CSM interventions have not been routinely used in patients with IBD. Because there is a significant overlap between symptoms of irritable bowel syndrome and those of IBD, we hypothesized that adapting the CSM intervention to a population of individuals with IBD may also be effective (CSM-IBD). However, this remains to be tested.

### Objectives

The objectives of this pilot randomized controlled trial are to (1) determine the feasibility and acceptability of study procedures (recruitment, randomization, and data and sample collection) and CSM-IBD; (2) compare CSM-IBD to usual care on changes from baseline to 3 months post intervention in quality of life and daily symptoms (fatigue, sleep disturbance, psychological distress, gastrointestinal symptoms) among IBD patients and (3) explore the association of socioecological factors (age, sex, race, ethnicity, or diet), clinical phenotype (medications, disease distribution, and disease activity), and biological signatures (microbiome and calprotectin) with symptoms at baseline and response to intervention (post intervention).

## Methods

### Study Design

The proposed study is a 2-arm pilot randomized controlled trial that follows the Consolidated Standards of Reporting Trials and Standard Protocol Items: Recommendations for Interventional Trials guidelines [30,31]. In a 2:1 ratio, 54 participants will be randomized (intervention=36 participants; usual care=18 participants). This randomization scheme was selected to maximize data collection and information regarding the intervention group. The intervention will take place over 8 sessions. The participants will be blinded to the study hypotheses. Web-based self-reported questionnaires will be used to reduce detection bias.

### Theoretical Framework

**Figure 1:**
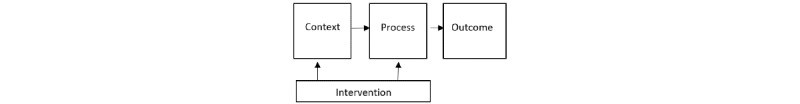
Individual and Family Self-Management Theory.

This study is guided by the Individual and Family Self-Management Theory (IFSMT) [32]. The IFSMT has 3 dimensions: context, process, and outcomes. It posits that interventions can influence the context and process (Figure 1). The context includes not only the individuals with IBD but also the complexity of the condition or treatment, individual factors (eg, age and sex), and social determinants of health such as food access and environmental settings, which can influence self-management skills and the ability to self-manage. In the IFMST, the process includes knowledge and beliefs (patient activation), self-regulation skills and abilities, and social facilitation and support (see Table 1 for the IFSMT process and CSM-IBD content). The IFSMT process of knowledge and beliefs includes Bandura self-efficacy theory, in which the intervention enhances self-efficacy (an individual’s belief in their capacity to execute behaviors) and thus improves self-management [33,34]. Specific strategies and approaches used within the intervention are based on the expansive literature on CBT [35,36]. By impacting both the context and process, the intervention can influence the outcome to decrease symptoms and improve quality of life.

**Table 1:** IFSMT^a^ self-management processes and CSM-IBD^b^ content.

IFSMT process	IFSMT description	CSM-IBD content
Knowledge and beliefs	Factual information Self-efficacy Goal congruence	“When to contact your provider” Introduction to strategies Explanation of self-management Personalized goals
Self-regulation skills and abilities	Goal-setting, self-monitoring, reflective thinking Decision-making, planning and action Self-evaluation Patient activation	Abdominal breathing Alternative thinking Passive or active progressive muscle relaxation Cognitive distortions Sleep patterns, sleep hygiene Pain management
Social facilitation	Social influence Support	Eating out Travel

^a^IFSMT: Individual and Family Self-Management Theory.

^b^CSM-IBD: comprehensive self-management for inflammatory bowel disease.

### Study Population

Participants are individuals seeking care for their IBD at the University of Washington (UW). Inclusion criteria include participants who (1) have a health care provider diagnosis of IBD as reported in the electronic medical record, (2) report at least 2 current, active symptoms (Short Inflammatory Bowel Disease Questionnaire [SIBDQ] equal or less than 4 [“some of the time”] on any 2 items), (3) are 18 to 75 years old, and (4) are able to read and write in English. Participant exclusion criteria include (1) pregnant individuals, (2) significant co-occurring mental or physical conditions that would impact study participation, (3) surgery or hospitalization within the past month, or (4) living outside of Washington State.

### CSM Intervention

The 8-session CSM includes patient education about self-management of symptoms using CBT, relaxation training, and diet counseling. The intervention will be delivered digitally with skill videos, readings, and homework, as well as a weekly phone call or Zoom check-in with a nurse trained in CBT. For each session, participants will review information and practice skills on the session topic. The content for each session is outlined in Table 2. The weekly phone calls are designed to provide accountability and support for achieving the session goals and to enable individuals to ask questions about the content. The estimated time of completion is 8 weeks; however, participants will have 12 weeks to complete the sessions to allow for unexpected events. Intervention fidelity will be monitored using fidelity checklists and through the evaluation of a random selection of 20% of participants.

Also of note, the original CSM intervention was administered as a paper workbook with weekly hour-long sessions with a trained advanced practice nurse. To improve accessibility and feasibility, we have adapted the intervention to be administered as a web-based intervention with weekly phone call check-ins with a CBT-trained nurse. This unique approach expands the feasibility and reach of the intervention by enabling patients who may live in rural or underserved areas to access the intervention content. To be appropriate for the IBD population, the session 1 disease content was modified to include the role of inflammation, the importance of endoscopic remission, and information on when to contact their health care provider. The intervention content was reviewed by patients and health care providers.

**Table 2:** Comprehensive self-management for inflammatory bowel disease (CSM-IBD) by session.

Session	Content
Introduction and tracking symptoms session	“When to contact your provider” Introduction to IBD^a^ Introduction to strategies and self-management Personalized goals and social support
Abdominal breathing and stress session	Role of stress Abdominal breathing
Problem-solving and travelling session	Alternative thinking or problem-solving Travelling
Healthy thought patterns session	Healthy thought patterns Automatic thoughts
Sleep and physical intimacy session	Sleep tracking Sleep hygiene Physical intimacy
Pain management and quick relaxation session	Pain management Passive or active progressive muscle relaxation
Healthy eating, tracking, and trigger foods session	Tips for healthy eating Eating out
Comprehensive plan session	Developing a comprehensive plan

^a^IBD: inflammatory bowel disease.

### Usual Care

The intervention group will be compared to a usual care group of participants who meet the same eligibility criteria. Due to a current lack of standardization in managing IBD within the clinic setting, a usual care patient group most accurately reflects current practice.

### Study Outcomes

#### Feasibility Outcomes

Feasibility will be evaluated for study procedures (recruitment, randomization, and data and sample collection) and the CSM-IBD intervention.

1. Recruitment: the number and percentage of participants prescreened, approached, screened, and enrolled.
2. Randomization: we will assess dropout rates between the CSM-IBD and usual care.
3. Data collection: the quality of data will focus on the data completeness of questionnaires; missing data will be reported as a percentage of missing data and the time point at which missing data occurred. Bivariate comparisons will be made between individuals with and without missing data. Information on sample collection time, transit time, and processing time will be obtained.
4. Sample collection: we will report how many participants collected biological samples on the planned date and the length of time from sample collection to arrival at the laboratory.
5. Intervention feasibility: measured with the Feasibility of Intervention Measure, in which participants respond to 4 questions on a 5-point Likert scale from 1=completely disagree to 5=completely agree at the end of the intervention [37].

#### Acceptability Outcomes

A semistructured interview guide will be used to assess the acceptability of study procedures as well as the acceptability and satisfaction with the CSM-IBD. For instance, questions will probe for clarity of instructions, the burden of study procedures, and satisfaction with intervention content and delivery. Transcripts from participant interviews will be coded by 2 individuals using open coding methods to evaluate intervention acceptability. The use of CSM-IBD strategies will be assessed at 3 months post intervention by asking participants how often they use each strategy, with response options as: not at all or rarely, occasionally (at least 1 day a week), often (at least 2 days a week), very often (at least 4 days a week), or almost always [38]. Participants will complete the Acceptability of Intervention Measure, which includes 4 questions rated on a 5-point Likert scale from 1=completely disagree to 5=completely agree [37]. Satisfaction will also be measured with the 8-item Client Satisfaction Questionnaire [39].

#### Primary Outcomes

##### Quality of Life

The SIBDQ is a 10-item questionnaire that evaluates the quality of life. Participants respond on a 7-item Likert scale from all the time to none of the time. Total score range: 10 to 70 [40].

##### Symptoms

Symptoms are measured using a numeric rating scale where participants report the severity of the symptom from 0=not present to 10=worst possible. Symptoms include abdominal pain, anxiety, bloating, constipation, depression, diarrhea, fatigue or tiredness, passing gas, sleepiness during the day, stress, and urgency. See Multimedia Appendix 1 for a copy of the IBD Symptom Scale.

#### Secondary Outcomes

1. Process variables [41,42]
  Self-Efficacy for Managing Chronic Disease: a 6-item self-report scale. Participants report ranging from not at all confident to totally confident [21].
  Index of Self-Regulation: a 9-item scale to measure an individual’s level of self-regulation [43].
  Patient Activation Measure: a 13-item scale that assesses patients’ beliefs, knowledge, and confidence in managing their health [44,45].
2. Biological signatures: stool samples will be collected by participants and stored immediately in home freezers (–20 °C) before transport to the UW School of Nursing Biobehavioral Laboratory. Samples will be stored at –80 °C until batch processing.
  Gut microbiome: fecal microbial communities will be characterized using metagenomic shotgun sequencing on the Illumina MiniSeq or NextSeq platforms (Illumina, Inc) [46,47].
  Fecal calprotectin: fecal calprotectin is a biomarker used to measure inflammation in the gut and will be assessed using an enzyme-linked immunosorbent assay [48].
3. Socioecological factors were selected based on the National Institute for Nursing Research’s common data elements and include sex, gender, age, race or ethnicity, education level, employment, marital or partner status, household size, and neighborhood (urban or rural, zip code). Dietary intake can influence the composition of the gut microbiome and symptoms [49]. At the time of the stool collection, subjects will complete a detailed 3-day food record (Fred Hutchinson Cancer Research Center Nutrition Assessment).
4. The clinical phenotype will be obtained from the electronic medical record: type of disease, time since diagnosis, history of bowel surgery, disease distribution (measured by the Montreal Classification), medications, and disease activity (Harvey Bradshaw Index for Crohn Disease [50] and Simple Colitis Activity Index for Ulcerative Colitis [51]; <5 indicates remission).
5. Health care use will be evaluated from the electronic medical record and patient self-report, including emergency department use, hospitalizations, outpatient visits, laboratory tests, imaging, and procedures, as well as providers’ nonclinical time spent managing patients.

### Participant Timeline

Multimedia Appendix 2 presents the schedule of enrollment, interventions, and assessments based on the Standard Protocol Items: Recommendations for Interventional Trials guidelines. All procedures will occur remotely; data collection will occur digitally through REDCap (Research Electronic Data Capture; Vanderbilt University), a secure Health Insurance Portability and Accountability Act (HIPAA)–compliant database [52,53]. Individuals meeting eligibility criteria and interested in the study will be asked to sign a consent form and HIPAA form prior to participating in the study. All participants will complete baseline questionnaires (socioecological factors and disease information). Participants will collect a stool sample at home using a stool sample collection kit (instructions and researcher contact information will be provided); a 3-day food record will be collected starting 2 days before the stool sample collection. The stool samples will be placed in home freezers and returned to the School of Nursing Biobehavioral Laboratory. Participants will be provided with ice packs, a Styrofoam mailing box, and return labels. Stool samples will be stored at –80 °C until batch processing.

Following baseline data collection, participants will be randomly assigned to the 8-session CSM-IBD or usual care. Immediately post intervention, participants will complete questionnaires, collect a stool sample, and report a 3-day food record. Three months post intervention, participants will complete questionnaires and participate in a qualitative data collection interview. In addition, participants will report whether or not they are continuing the self-management skills learned in the intervention. Semistructured interviews will be conducted to understand the participants’ perspective on procedural burden and suggestions to improve the intervention, including satisfaction with the intervention (see Multimedia Appendix 3). Interviews will be recorded and transcribed. Participants will be compensated for each assessment completed (baseline, immediately post intervention, and 3 months post intervention). To increase retention, participants in the usual care group will be provided access to the intervention upon study completion. Participants can continue the study despite not completing all data collection assessments and all intervention sessions.

### Sample Size

Based on recommendations for preliminary studies [54], we aim for data on 54 participants (36 intervention and 18 usual care). The sample size is appropriate for assessing feasibility and acceptability (aim 1). For aim 2, we will be able to detect fairly large effect sizes of 0.81 with 80% power. For aim 3, there will be 80% power to detect a correlation of 0.45. As a pilot study, the project is not powered to detect differences or correlations unless they are large.

### Recruitment

#### Overview

Participant recruitment will take place at the UW Inflammatory Bowel Disease Clinics. The UW sees a large number of patients with IBD and serves as a tertiary care clinic for patients with IBD in the Washington, Oregon, Alaska, Montana, and Idaho regions. Recruitment may occur in three different settings: (1) in a clinic, (2) virtually (via telephone, email, or mail), and (3) by posting on the Institute of Translational Health Sciences website. All individuals will be informed that participating or not participating will not influence the care that they receive.

#### In a Clinic

Weekly prescreening will occur to determine if participants with scheduled IBD clinic appointments may be eligible for the study. The goal of prescreening is to enable purposeful sampling of patients based on demographic (eg, age and race) and disease characteristics (eg, disease activity). Individuals who are eligible will receive a flyer containing the study purpose and contact information from either their health care provider or study staff (depending on feasibility and current COVID-19 restrictions). Individuals will have time to review the study information. Participants can call study staff or complete a REDCap survey to express their interest and complete screening questions.

#### Virtually

The team may contact potential participants via phone, email, or mail if in-person recruitment becomes difficult or not feasible. All contact will follow the UW’s institutional review board guidelines for cold contact recruitment. Those expressing interest in the study will call study staff for more information and then complete additional screening questions.

#### Institute of Translational Health Sciences Website

Information regarding the study will be posted on the UW Institute of Translational Health Sciences website. Interested individuals can complete the contact form to be contacted by study staff and can complete screening questions digitally.

In all instances, the purpose of the study, study design, and length of time to complete the study will be discussed. To assess participants’ understanding of study procedures, individuals will be asked to briefly describe the study. Interested individuals will complete additional screening questions to confirm whether patients meet the specified inclusion and exclusion criteria. Screening questionnaires may be completed over the phone or electronically via REDCap, based on patient preference. Those meeting inclusion or exclusion criteria will be invited to participate in the study, sign a consent form, and begin data collection. Throughout the entire process of informed consent, from the first contact with the patient, the potential participants are able to ask any questions or delay making a decision. This screening and recruitment process will continue until the target sample size has been reached.

### Intervention Allocation

A computer adaptive randomization procedure using a modification of the minimization method proposed by Pocock and Simon [55] will be used to balance groups based on SIBDQ at baseline. Once the data are entered into the computer, the computer program will return the participants’ group assignment.

### Statistical Analysis

#### Feasibility

The number and percentage will be calculated for participants who were prescreened, approached, screened, and enrolled (recruitment). We will assess dropout rates between the CSM-IBD and usual care (randomization). Quality of data will focus on the data completeness of questionnaires; missing data will be reported, and bivariate comparisons will be made between individuals with and those without missing data (data collection). We will report how many participants collected biological samples within the desired timeframe (sample collection). The mean and SD will be reported for the Feasibility of Intervention Measure (intervention feasibility).

#### Acceptability

Transcripts from participant interviews will be coded by 2 individuals using open coding methods (acceptability and satisfaction). In addition, numbers and percentages will be reported for participants who continue to use self-management strategies 3 months post intervention.

#### Primary Outcomes

Data from the 2 follow-up time points (immediate and 3 months post intervention) will be analyzed together using linear mixed models for each of the outcomes. For example, one model will have a change in the quality of life from baseline to immediate and 3 months post intervention as the outcomes, subject as a random effect, time (immediate or 3 months) as a within-subject fixed factor, and treatment group as an across-subject fixed factor. Baseline quality of life will be included as a covariate in the model, as will any baseline variables that differ between the 2 treatment groups. The parameter for the treatment group factor will provide an estimate of the treatment effect, that is, the difference between the treatment groups in mean change in the quality of life, and a 95% CI on the estimated difference will be computed. We hypothesize participants in the CSM-IBD will report a greater reduction in symptoms compared to those in usual care. A second model will also include an interaction between group and time to test whether the treatment effect is different at the 2 post intervention times. Additional analyses will estimate treatment effects separately for men and women.

#### Secondary Outcomes

Analyses of microbiome data will use 3 summary measures—Shannon diversity, richness, and *Firmicutes/Bacteroidetes* ratio—as well as the relative abundance of bacterial families (eg, family Ruminococcaceae) and genera and will control for the read count. For diet, we will focus on protein and fiber intake, as our previous work has indicated these may be important dietary intake components related to the gut microbiome. First, we will examine the associations between socioecological, clinical phenotype, and biological signatures with symptoms at baseline in the total sample using Pearson correlation or Spearman rank correlation based on measure distributions. Scatter plots will be used to visually display these associations. Second, we will examine associations between socioecological, clinical phenotypes, and biological signatures at baseline and response to intervention in the intervention group. Response to intervention is conceptualized as an increase of 8 points on the SIBDQ scale (quality of life) [40] or a 50% decrease from baseline in the GI symptom score [27]. Among the intervention group, we will use the *t* test and chi-square test to compare socioecological factors, clinical phenotypes, and biological signatures among patients with and without a response to treatment.

### Monitoring

This study has been classified as a minimal-risk study. The principal investigator has the responsibility for study oversight, including patient safety and data quality, as well as submitting reports to the funder and the institutional review board. Due to the low-risk nature of the CSM-IBD, we do not anticipate any adverse events or serious adverse events. The data safety and monitoring team will meet quarterly to review the study. The team will monitor accrual, withdrawals, data quality, timeliness of data submission, protocol compliance, and the types and frequency of adverse events and serious adverse events. No interim analysis or stopping rules are currently planned. However, if any adverse events or serious adverse events are reported, the data safety and monitoring team will meet within a week to discuss the event and any potential changes needed to the study procedures.

### Ethics and Dissemination

#### Data Management

Study data will be collected and stored on REDCap. Participants will be assigned a unique study identification number. Only study members will have access to the data through a password-protected system and will have access to the least amount of data required for their position using limitations in REDCap user rights. The study will use ongoing quality control procedures during data collection, storage, and processing, including the use of the REDCap data quality module.

#### Ethics Approval

The institutional review board at the UW approved this protocol (STUDY 00015210) and determined the study to be minimal risk. This study is registered in ClinicalTrials.gov (NCT05651542). Any amendments to the study will be reviewed by the institutional review board and subsequently updated in the ClinicalTrials.gov registry. Participants in the usual care group will be offered access to the CSM-IBD intervention after they complete the 6-month data collection.

#### Dissemination

Study findings will be disseminated through presentations and publications to clinicians and researchers. Findings will be reported to the community through lay presentations and social media. Individuals will be eligible for authorship based on the International Committee of Medical Journal Editors’ recommendations. The study will provide the basis for future research on self-management interventions. The findings of the study will be used to refine the intervention and develop a full-scale trial with additional recruitment sites throughout diverse geographical regions.

### Patient Involvement

This study is informed by a patient advisory group that was convened for the purpose of providing advice and oversight for the study.

## Results

This pilot randomized controlled trial was funded by the National Institute of Nursing Research (K23NR020044) in April 2022. Recruitment began on February 20, 2023. As of April 2023, we have enrolled 4 participants. We expect the study to be completed by March 2025.

## Discussion

### Expected Findings

This project is focused on evaluating the feasibility, acceptability, and preliminary efficacy of a CSM intervention for improving the quality of life and symptoms among individuals with IBD. We expect that overall participants will find the intervention acceptable and feasible, as mixed methods systematic review reported that greater than 75% of participants in IBD-related telehealth and mobile health interventions reported satisfaction with the intervention [56]. We also anticipate receiving feedback from participants on ways to improve the intervention.

Further, we expect to find preliminary signals of efficacy. A similar CSM intervention implemented in individuals with IBS showed reductions in anxiety, depression, abdominal pain, fatigue, and extraintestinal pain and improvements in sleep and quality of life [25-29]. A recently published study of a digital health program in IBD reported promising improvements in stress and energy levels, with a participant retention rate of 40% [57]. We hope that our approach of incorporating weekly check-ins will lead to higher retention rates. Findings from the study will be used to refine the intervention and develop a full-scale trial that will incorporate additional recruitment sites from diverse geographical regions. In future trials, we plan to collaborate with a variety of clinical practice settings, including community-based and academic settings.

### Strengths and Limitations

Individuals participating in a pilot self-management intervention may be more interested in or engaged in research and self-management than a general clinic population. Additionally, recruitment is currently focused on an academic medical setting, which may have a more medically refractory group of patients. Thus, expanding recruitment to other sites (eg, community sites) may assist with generalizability. To enhance its feasibility and reach, the intervention has been adapted into a web-based format.

### Future Directions

If CSM-IBD proves effective, further research into predictors of response to CSM would be warranted, including assessing the correlation with the microbiome, histology, or co-occurring illness (eg, depression and anxiety). Other future directions include consideration for implementation barriers and facilitators. Future work may consider multilevel interventions that can impact the health care system level to promote self-management and quality of life.

### Conclusions

The results of this study will inform the development of a full-scale clinical trial to examine the impact of CSM-IBD compared to usual care. If the intervention demonstrates acceptability and efficacy, it could be deployed in a variety of practice settings to improve patient self-management of IBD.

## Appendix

Multimedia Appendix 1 IBD Symptom Scale.

Multimedia Appendix 2 Schedule of enrollment, interventions, and assessments—SPIRIT figure. CSM-IBD: comprehensive self-management for inflammatory bowel disease; SIBDQ: Short Inflammatory Bowel Disease Questionnaire.

Multimedia Appendix 3 End of Study Interview Questions.

Multimedia Appendix 4 Peer-review report by the National Institute of Nursing Research Initial Review Group (National Institutes of Health, USA).

## Abbreviations

CBT: cognitive behavioral therapy
CSM: comprehensive self-management intervention
CSM-IBD: comprehensive self-management for inflammatory bowel disease
HIPAA: Health Insurance Portability and Accountability Act
IBD: inflammatory bowel disease
IFSMT: Individual and Family Self-Management Theory
SIBDQ: Short Inflammatory Bowel Disease Questionnaire
UW: University of Washington

## Data Availability

The data sets generated during this study will be deposited in a publicly available repository at study completion.
