# Supplementary material for: A Comprehensive Self-Management Intervention for Inflammatory Bowel Disease (CSM-IBD): Protocol for a Pilot Randomized Controlled Trial
Source: JMIR Res Protoc. 2023 Jun 7;12:e46307. doi: 10.2196/46307 (PMC10285620; doi:10.2196/46307)
Supplement: Multimedia Appendix 1 [file resprot_v12i1e46307_app1.docx]

# Appendix 1

## IBD Symptom Scale

**For each of the below symptoms, please indicate the extent to which you experienced each symptom over the past week.**

Abdominal pain or discomfort

| 0  (not present) | 1 | 2 | 3 | 4 | 5 | 6 | 7 | 8 | 9 | 10  (worst possible) |
| --- | --- | --- | --- | --- | --- | --- | --- | --- | --- | --- |

Anxiety

| 0  (not present) | 1 | 2 | 3 | 4 | 5 | 6 | 7 | 8 | 9 | 10  (worst possible) |
| --- | --- | --- | --- | --- | --- | --- | --- | --- | --- | --- |

Bloating

| 0  (not present) | 1 | 2 | 3 | 4 | 5 | 6 | 7 | 8 | 9 | 10  (worst possible) |
| --- | --- | --- | --- | --- | --- | --- | --- | --- | --- | --- |

Constipation

| 0  (not present) | 1 | 2 | 3 | 4 | 5 | 6 | 7 | 8 | 9 | 10  (worst possible) |
| --- | --- | --- | --- | --- | --- | --- | --- | --- | --- | --- |

Depressed/sad or blue

| 0  (not present) | 1 | 2 | 3 | 4 | 5 | 6 | 7 | 8 | 9 | 10  (worst possible) |
| --- | --- | --- | --- | --- | --- | --- | --- | --- | --- | --- |

Diarrhea

| 0  (not present) | 1 | 2 | 3 | 4 | 5 | 6 | 7 | 8 | 9 | 10  (worst possible) |
| --- | --- | --- | --- | --- | --- | --- | --- | --- | --- | --- |

Difficulty falling asleep

| 0  (not present) | 1 | 2 | 3 | 4 | 5 | 6 | 7 | 8 | 9 | 10  (worst possible) |
| --- | --- | --- | --- | --- | --- | --- | --- | --- | --- | --- |

Fatigue/tiredness

| 0  (not present) | 1 | 2 | 3 | 4 | 5 | 6 | 7 | 8 | 9 | 10  (worst possible) |
| --- | --- | --- | --- | --- | --- | --- | --- | --- | --- | --- |

Joint pain

| 0  (not present) | 1 | 2 | 3 | 4 | 5 | 6 | 7 | 8 | 9 | 10  (worst possible) |
| --- | --- | --- | --- | --- | --- | --- | --- | --- | --- | --- |

Nausea

| 0  (not present) | 1 | 2 | 3 | 4 | 5 | 6 | 7 | 8 | 9 | 10  (worst possible) |
| --- | --- | --- | --- | --- | --- | --- | --- | --- | --- | --- |

Passing gas/flatulence

| 0  (not present) | 1 | 2 | 3 | 4 | 5 | 6 | 7 | 8 | 9 | 10  (worst possible) |
| --- | --- | --- | --- | --- | --- | --- | --- | --- | --- | --- |

Sleepiness during the day

| 0  (not present) | 1 | 2 | 3 | 4 | 5 | 6 | 7 | 8 | 9 | 10  (worst possible) |
| --- | --- | --- | --- | --- | --- | --- | --- | --- | --- | --- |

Stress

| 0  (not present) | 1 | 2 | 3 | 4 | 5 | 6 | 7 | 8 | 9 | 10  (worst possible) |
| --- | --- | --- | --- | --- | --- | --- | --- | --- | --- | --- |

Urgency

| 0  (not present) | 1 | 2 | 3 | 4 | 5 | 6 | 7 | 8 | 9 | 10  (worst possible) |
| --- | --- | --- | --- | --- | --- | --- | --- | --- | --- | --- |

Waking up during the night

| 0  (not present) | 1 | 2 | 3 | 4 | 5 | 6 | 7 | 8 | 9 | 10  (worst possible) |
| --- | --- | --- | --- | --- | --- | --- | --- | --- | --- | --- |

**In the past week, have you had the following:**

Blood in your stool? Yes/No

Mucus in your stool? Yes/No
