# Supplementary figures and images for: A Comprehensive Self-Management Intervention for Inflammatory Bowel Disease (CSM-IBD): Protocol for a Pilot Randomized Controlled Trial
Source: JMIR Res Protoc. 2023 Jun 7;12:e46307. doi: 10.2196/46307 (PMC10285620; doi:10.2196/46307)

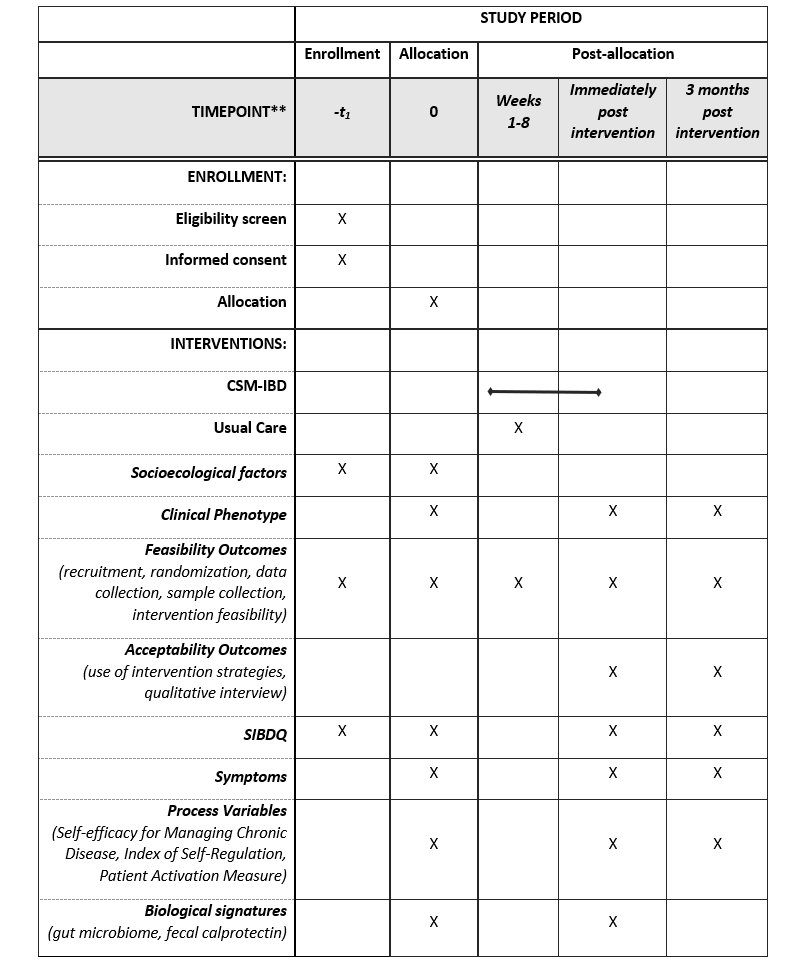

Supplement: Multimedia Appendix 2 [file resprot_v12i1e46307_app2.png]
