# Supplementary material for: A Comprehensive Self-Management Intervention for Inflammatory Bowel Disease (CSM-IBD): Protocol for a Pilot Randomized Controlled Trial
Source: JMIR Res Protoc. 2023 Jun 7;12:e46307. doi: 10.2196/46307 (PMC10285620; doi:10.2196/46307)
Supplement: Multimedia Appendix 3 [file resprot_v12i1e46307_app3.docx]

# Appendix 3

## End of Study Interview

Thank you so much for participating in this research study. The purpose of this conversation is to obtain your feedback on the study and learn ways that we can improve the study for others. Before we begin, is it okay if I record our conversation?

- How easy was it to participate in the study?
- Were the study instructions clear?
- Did the process of completing all questionnaires seem burdensome?
- Was there anything unusual about any of the questions?
- How was the process of stool sample collection? Anything that could be improved?
- Is there anything you would have changed about the study?
- What did you enjoy about the study?
- What did you not enjoy about the study?
- Is there anything else you would like us to know?

*Intervention Only*

- Would you recommend this intervention to a friend? Why or why not?
- Are you satisfied with the intervention? Why or why not?
- What was the best part of the intervention? What was the worst part?
- How would you change the intervention to make it more usable?
