# Supplementary material for: A Comprehensive Self-Management Intervention for Inflammatory Bowel Disease (CSM-IBD): Protocol for a Pilot Randomized Controlled Trial
Source: JMIR Res Protoc. 2023 Jun 7;12:e46307. doi: 10.2196/46307 (PMC10285620; doi:10.2196/46307)
Supplement: Multimedia Appendix 4 [file resprot_v12i1e46307_app4.pdf]

SUMMARY STATEMENT
(Privileged Communication)

PROGRAM CONTACT:
David Banks
301-496-9558
banksdh@mail.nih.gov

Release Date: 11/23/2021
Revised Date:

Application Number: 1 K23 NR020044-01A1
Formerly: 1K23NR020044-01

Principal Investigator

KAMP, KENDRA JOY

Applicant Organization: UNIVERSITY OF WASHINGTON

Review Group: NRRC (82)
National Institute of Nursing Research Initial Review Group

Meeting Date: 10/21/2021
Council: JAN 2022
Requested Start: 04/01/2022

RFA/PA: PA20-206
PCC: DXYDB
Dual PCC: RAJ DUAL
Dual IC(s): DK

Project Title: A Comprehensive Self-Management Intervention for individuals with Inflammatory Bowel Disease
SRG Action: Impact Score:20
Next Steps: Visit https://grants.nih.gov/grants/next_steps.htm
Human Subjects: 30-Human subjects involved - Certified, no SRG concerns
Animal Subjects: 10-No live vertebrate animals involved for competing appl.
Gender: 1A-Both genders, scientifically acceptable
Minority: 1A-Minorities and non-minorities, scientifically acceptable
Age: 3A-No children included, scientifically acceptable

Project Year	Direct Costs Requested	Estimated Total Cost
1	117,100	126,468
2	117,100	126,468
3	117,100	126,468
TOTAL	351,300	379,404

ADMINISTRATIVE BUDGET NOTE: The budget shown is the requested budget and has not been adjusted to reflect any recommendations made by reviewers. If an award is planned, the costs will be calculated by Institute grants management staff based on the recommendations outlined below in the COMMITTEE BUDGET RECOMMENDATIONS section.

1K23NR020044-01A1 Kamp, Kendra

RESUME AND SUMMARY OF DISCUSSION: This report summarizes the merit review of a resubmitted application for a Mentored Patient-Oriented Research (POR) Career Development Award from Dr. Kendra Kamp, an Assistant Professor at the University of Washington (UW), who requests three years of support to become an expert in self- and symptom-management among individuals with inflammatory bowel disease (IBD). Based on a Comprehensive Self-Management Intervention (CSMI) shown to reduce symptoms among persons with IBS, she proposes to evaluate feasibility, acceptability, and preliminary efficacy of the CSMI adapted for those with IBD; she will also examine associations of symptoms with demographic characteristics, biological signature, and clinical phenotypes. This resubmission is very responsive to prior critiques by including additional microbiome analyses training, justified sample size, clarified timeline, and additional satisfaction measures; however, the analytic plan for qualitative data remains underdeveloped. The accomplished and productive applicant and her assembled team of mentors renowned for their expertise in IBD and IBS continue to be major strengths of the resubmission, as does the well-resourced environment at UW. In response to previous reviewer comments, the target population is restricted to patients with IBD to be distinct from that of her mentors, thereby increasing the likelihood of independence. Additional clarity about the proposed hands-on microbiome training and the extraction of DNA/RNA from fecal samples justify these Career Development activities, although the training goal to increase diversity of research participants does not have associated objectives or activities. The revised Research Plan features rigorous methodology and a well-designed clinical trial. However, plans for recruitment and retention of individuals from racial and ethnic minority groups and assuring treatment fidelity are underdeveloped, and rationale for usual care control group, 2:1 randomization, timing of data collection, and some exclusion criteria are unclear. Reviewers agree that successfully completing the specific aims will advance the field of IBD symptom management and could significantly impact clinical practice and this improved application is ranked as outstanding for its scientific and technical merit.

DESCRIPTION (provided by applicant): Individuals with inflammatory bowel disease (IBD) experience a high burden of symptoms including abdominal pain, bloating, fatigue, sleep disturbances, anxiety, and depression. Yet, few self-management interventions exist to reduce symptoms in this population. This proposed study will adapt an existing Comprehensive Self- Management Intervention (CSM) with demonstrated efficacy in an irritable bowel syndrome population into a population of individuals with IBD (CSM-IBD). The long-term training goal of this project is to assist Dr. Kamp in becoming an independent investigator with a program of research focused on improving self- and symptom- management among individuals with IBD. As such, Dr. Kamp has training competencies to increase knowledge and skills in advanced training in conducting randomized controlled trials, longitudinal data management, processing and analysis of microbiome data and leading interdisciplinary teams. The Specific Aims are to: 1) determine the feasibility and acceptability of study procedures (recruitment, randomization, data and sample collection) and the CSM-IBD; 2) compare the CSM-IBD intervention to usual care on changes from baseline to 3 months post-intervention in quality of life and daily symptoms (fatigue, sleep disturbance, psychological distress, and GI symptoms); and 3) explore the association of symptoms with socioecological factors (age, sex, race/ethnicity, diet), clinical phenotype (medications, disease distribution), and biological signatures (microbiome, calprotectin) with symptoms at baseline and response to intervention (immediately post- intervention). The expected outcome will be preliminary feasibility data to inform a future R01 study which will examine the efficacy of the CSM-IBD. To accomplish the research aims and training goals, an interdisciplinary mentorship team has been assembled with expertise in symptom science, IBD, psychology, interdisciplinary research, RCTs, gut microbiome, statistics, and data management. The team, along with the vast array of resources available at the University of Washington, are well-suited to transition Dr. Kamp into an independent investigator. This research is significant because it will address an unmet need regarding self-management among individuals with IBD and has the potential
